# Executive Function Buffers the Association between Early Math and Later Academic Skills

**DOI:** 10.3389/fpsyg.2017.00869

**Published:** 2017-05-30

**Authors:** Andrew D. Ribner, Michael T. Willoughby, Clancy B. Blair, The Family Life Project Key Investigators

**Affiliations:** ^1^Department of Applied Psychology, New York University, New YorkNY, United States; ^2^RTI International, Research Triangle EastNC, United States; ^3^Center for Developmental Science, University of North Carolina at Chapel Hill, Chapel HillNC, United States; ^4^Department of Human Development and Family Studies, Pennsylvania State University, University ParkPA, United States

**Keywords:** executive function, math achievement, reading development, elementary school children, academic achievement, moderation

## Abstract

Extensive evidence has suggested that early academic skills are a robust indicator of later academic achievement; however, there is mixed evidence of the effectiveness of intervention on academic skills in early years to improve later outcomes. As such, it is clear there are other contributing factors to the development of academic skills. The present study tests the role of executive function (EF) (a construct made up of skills complicit in the achievement of goal-directed tasks) in predicting 5th grade math and reading ability above and beyond math and reading ability prior to school entry, and net of other cognitive covariates including processing speed, vocabulary, and IQ. Using a longitudinal dataset of *N* = 1292 participants representative of rural areas in two distinctive geographical parts of the United States, the present investigation finds EF at age 5 strongly predicts 5th grade academic skills, as do cognitive covariates. Additionally, investigation of an interaction between early math ability and EF reveals the magnitude of the association between early math and later math varies as a function of early EF, such that participants who have high levels of EF can “catch up” to peers who perform better on assessments of early math ability. These results suggest EF is pivotal to the development of academic skills throughout elementary school. Implications for further research and practice are discussed.

## Introduction

Children’s success in schooling has long been a central focus of research, policy, and practice. In December 2015, the Every Student Succeeds Act was signed into law at a time that marked all-time high graduation rates and low dropout rates in the United States. The Every Student Succeeds Act, in concert with the Common Core State Standards, were meant to improve graduation rates and further minimize student dropout. Yet, still 6.5% of all students entering high school, and 11.6% of students who are born to families from the lowest income quartile drop out of high school. Further, these dropout rates are highest in the American South, and in rural areas across the country (U.S. Department of Education, National Center for Education Statistics, 2016). At the same time, many other countries experience large proportions of students dropping out of high school, and only some 33% of students in OECD countries enroll in postsecondary education. Importantly, while there has been some improvement in secondary school attainment internationally, there is also a marked degree of stability in secondary school dropout (Lamb et al., 2010; OECD, 2016). Decades of research in the United States and abroad has suggested a substantial amount of student dropout is attributable to school, teacher, and classroom characteristics (e.g., Ehrenberg and Brewer, 1994; Rumberger and Thomas, 2000; Koedel, 2008; Hanushek et al., 2008); however, it remains important to attend to individual-level skills that predict and promote academic success so as to develop effective ways to enhance school achievement.

Extensive evidence has suggested that early academic skills are a robust indicator of later achievement (Duncan et al., 2007). In many cases and to a large extent, high-quality early learning experiences may account for the early academic aptitude of a young child (e.g., Melhuish et al., 2008), but an outstanding question remains: What individual characteristics makes a successful young reader or mathematician? Given that there is extensive variation in early learning experiences—which shape early academic skills—what is the contribution of individual factors to later academic success? We seek to better understand whether early academic skills are as deterministic of later academic ability as prior investigations might suggest (e.g., Duncan et al., 2007). The goal of the present study is to understand whether individual cognitive skills may compensate for lower levels of early preparedness of academic success in late elementary school in a sample of students living in low-income families in two rural areas of the United States. Multiple candidate predictors were tested for their unique contribution to 5th grade math and reading skills. We focus primarily on executive function (EF) prior to school entry and examine its predictive validity while simultaneously considering a number of well-established predictors of school outcomes including subject specific prekindergarten (PreK) academic knowledge, and other indicators of cognitive functioning, including vocabulary, IQ and processing speed. Beyond understanding robust predictors of later academic ability, controlling for early academic ability, we also sought to test whether PreK cognitive abilities might be compensatory, that is, help a child “catch up” to their peers if they begin schooling with relatively low level of academic ability.

### Background

For a child to succeed in modern society, they must be a successful reader. The ability to read is foundational for nearly all school-based learning and undergirds opportunities for academic and vocational success. Importantly, the development of reading has been characterized as a process in which the child must transition from “learning to read” to “reading to learn” (Center for Public Education, 2015). For over 20 years, the United States has made it a national priority to make every child a proficient reader by the end of 3rd grade, and yet still over 50% of children test below the level of proficiency on reading assessments as recently as 2015 (U.S. Department of Education, Institute of Education Sciences, National Center for Education Statistics, 2015). Crucially, an even greater proportion of children from low-income families (as measured by eligibility for free/reduce price lunch) test below proficient on reading assessments. This is important because nearly three-quarters of students who test below proficient in 3rd grade remain below proficient in high school (Shaywitz et al., 1992), and are four times more likely to drop out of high school than their peers who test proficient (Hernandez, 2011). More research examining early individual-level predictors of later reading ability and reading difficulty is needed.

As with reading, the late elementary grades appear to be an important transition time for the development of mathematics ability: Children who fail a math course in 6th grade have a 60% chance of dropping out of high school (Balfanz et al., 2007). The National Mathematics Advisory Panel (2008) stated in their report that in order to be prepared for high school graduation and college attendance, students should prove a firm understanding of topics covered in Algebra 2 by the time they are eligible for high school graduation. In order to be on track to succeed in Algebra 2, students should be enrolled in Algebra 1 by 8th grade, or when children are around 14 years old. As with reading, however, national assessments reveal less than 50% of children perform above the proficient level in math, and over 75% of students from low-income homes perform below the proficient level (U.S. Department of Education, Institute of Education Sciences, National Center for Education Statistics, 2015).

The importance of achievement in elementary school academics is not simply related to later academic attainment. Several studies have found that test scores prior to high school are positively associated with labor market outcomes, including income and employment, even when analyses control for educational attainment (Rose, 2006). Such studies have found positive associations between both reading and math achievement and labor market outcomes over and above motivation and intelligence as early as when children are age seven, or around 3rd grade (Ritchie and Bates, 2013).

What, then, differentiates a successful elementary school reader and mathematician from an unsuccessful one? Extensive evidence from the last decade has suggested that early skills predict later skills: the strongest and most robust predictor of a child’s later academic skills is their earlier academic skills. Duncan et al. (2007) reported in a meta-analysis of six nationally-representative datasets of three countries that math and reading skills at kindergarten entry robustly predicted high school math and reading skills net of background characteristics and socioemotional skills. These findings have been replicated and extended to suggest that early academic skills are important even for certain socioemotional skills in later years (Romano et al., 2010), and over a variety of time scales (Jordan et al., 2009). Collectively, these studies have tested PreK and kindergarten behavioral, cognitive, and socioemotional skills that predict scores on assessments of reading and math ability and suggested that, net of a broad host of covariates, there is a strong domain-specific stability of academic skills.

However, the development of academic skills does not occur in isolation: children are exposed to a multitude of academic settings that contribute to the promotion of math and reading skills. As such, intervention in the time between school entry and late elementary school could have an effect. Experimental studies have shown that curricular intervention in the early elementary years can result in improved domain-specific skills. However, the effects are limited. A meta-analysis of elementary school math intervention programs for typically performing students found that even the most successful intervention programs had a median effect size of +0.33 (Slavin and Lake, 2008). Similarly, highly effective reading intervention programs for children between kindergarten and 1st grade showed a median effect size of +0.22, and for children between 2nd and 5th grade, a median effect size +0.13. These findings suggest that there is only so much that can be done in domain-specific instructional settings to move the needle on academic ability between school entry and late elementary school.

A separate, though highly related literature has suggested that there are other classroom skills that may contribute to the development of math and reading skills during elementary years (Durlak et al., 2011). For years, there has been an interest in EF as a driving force of academic learning. EF comprises skills engaged in service of goal-directed behaviors, which include the ability to inhibit highly automatic or prepotent responses to stimulation, to store and manipulate information in working memory, and to flexibly shift the focus of attention among multiple relevant aspects of a given set of stimuli. EF skills are important for children’s learning, especially in their ability to attend to and integrate information taught in classroom settings, and have been implicated in the development of academic skills (Blair, 2002; Blair and Razza, 2007; Best et al., 2011). Further, there has been extensive evidence to suggest specific associations between EF and the development of each reading and math in elementary school.

A robust literature has indicated a relation between EF and reading skills throughout the academic lifespan. There is evidence that EF is related to early precursors to reading (Blair and Razza, 2007), and that the associations between EF and reading is present and largely invariant from when children are in elementary grades (e.g., when they are making the transition from “learning to read” to “reading to learn”) through high school years (Christopher et al., 2012). Though there is some question of directionality of influence (i.e., whether EF underlies the development of reading or whether successful reading improves EF), there are correlational studies which suggest children who have impaired reading abilities also have particularly weak EF skills (Carretti et al., 2009; Cutting et al., 2009), and that there is unique variance contributed to reading comprehension by EF, net of a host of other factors commonly associated with the development of reading comprehension (Sesma et al., 2009). As well, there is evidence from cognitive neuroscience that the development and change of brain structures that support EF parallels the process of reading acquisition (Cartwright, 2012).

The association between EF and math has been similarly well documented. There has been extensive correlational evidence to suggest EF contributes significant variance to success in math across a wide range of age groups, from preschool and kindergarten (Blair and Razza, 2007; Kroesbergen et al., 2009) through adulthood (Kalaman and Lefevre, 2007) and at intervening ages (Swanson, 2004; Männamaa et al., 2012). Additionally, a limited number of experimental and training studies have corroborated and added a directional component to the hypothesis that EF underlies the development of mathematical skills. For example, there is some evidence that training EF skills in early and middle childhood results in better numeracy and math reasoning skills (Fuchs et al., 2003; Kroesbergen et al., 2014). As with reading skills, there is also evidence that poor EF is often correlated with math learning disabilities (Clark et al., 2010; Toll et al., 2011; Willoughby et al., 2016).

Additionally, prior studies have suggested EF may interact with other early academic skills to moderate the association between early and later academic skills. Studies have shown through cross-lagged models that EF predicts change in math and reading skills over and above stability from PreK to kindergarten (Welsh et al., 2010), and a recent analysis from the current dataset found that the interaction of EF at age 4 with math in PreK moderated the strength of the association between math abilities in PreK and kindergarten (Blair et al., 2016). Similarly, a separate study found higher levels of EF skills in kindergarten related to faster growth of math skills in early elementary school (Lee and Bull, 2016). Together, these suggest that there may be a compensatory effect of EF: despite a high degree of stability between early academic skills and later academic skills (e.g., La Paro and Pianta, 2000; Duncan et al., 2007), there may be alternative mechanisms that could be leveraged to help students “catch up” throughout the elementary years.

### Present Study

The objective of the present study is to investigate the unique role of EF measured in early childhood in predicting academic achievement in late elementary school, an important transition time in children’s academic career. In particular, we are interested in the predictors of academic skills for students from predominantly low-income and rural (non-urban) areas of the United States. These students are at elevated risk for failure to complete high school and dropping out of school. We analyze data collected on children’s EF and math and literacy skills, along with other cognitive functions such as IQ, speed of processing, and receptive vocabulary prior to kindergarten entry, then assess math and literacy skills again when children are in 5th grade.

We pose two primary questions in the present study. First, we investigate predictors of academic skills in 5th grade. Our first hypothesis is that child EF measured prior to school entry will be uniquely associated with both later math and reading skills, even when controlling for cognitive functions and early math and reading with which EF is known to be associated. As such, we intend to estimate the amount of change in academic achievement attributable to EF above and beyond earlier academic knowledge and cognitive functioning. Second, we extend the analyses of Blair et al. (2016) to test whether there may be a compensatory effect of EF or other cognitive skills to 5th grade. That is, we investigate whether EF ability changes or moderates the association between early math and reading ability, measured in preK, and later math and reading ability measured in grade 5. It is expected that findings from this analysis will indicate an important mechanism through which children with lower levels of academic ability at school entry can “catch up” to their higher-achieving peers. As such, we hypothesize that children with high levels of EF at school entry will perform well on assessments of math and literacy in late elementary school, even if they had low levels of achievement at school entry.

## Materials and Methods

### Participants

Participants were recruited as a part of a prospective, longitudinal study. The Family Life Project (FLP) recruited children and their families from two distinct geographical areas of the United States with high rates of poverty. Three counties in eastern North Carolina and three in central Pennsylvania were selected to be indicative of the Black South and Appalachia, respectively. Children were recruited to be representative of one of the six counties in which families resided at the time of the child’s birth. Low-income families were oversampled in both states, and African American families were oversampled in North Carolina. Full details of the sampling procedure have been described elsewhere (Vernon-Feagans and Cox, 2013).

A total 1,292 families were recruited to take part in data collection when the child was 2 months of age, at which point they were formally enrolled in the study.

### Procedures

Demographic data were drawn from regularly scheduled home visits conducted over the course of time when children were 2 months old to 3 years old. EF data were drawn from direct assessment conducted during a home visit when children were 5 years old. Academic skills were measured prior to kindergarten entry (PreK) and in 5th grade. Assessments took place in school settings when possible, or in home settings in cases that children were not enrolled in center- or school-based care at any of the time points. Children were also assessed in school settings during kindergarten, 1st, 2nd, and 5th grades. A subset of children was also assessed in school settings during 3rd grade. Additionally, children were assessed in the home seven times between when children were 2 months and 5 years of age. Only data from the PreK, age 5, and 5th grade data collection time points are included in the present study.

### Measures

#### Executive Function (EF)

Executive function assessment comprised six tasks. All tasks were administered on an open spiral-bound notebook by a trained research assistant. These tasks are described in detail and evaluated elsewhere (Willoughby et al., 2010; Willoughby and Blair, 2011; Willoughby et al., 2012) and thus only abbreviated descriptions of each task are provided.

##### Working memory span (working memory)

Children were shown a line drawing of an animal and a color inside an image of a house and asked to keep both the animal and the color in mind, and to recall one of them (e.g., animal name) when prompted. Task difficulty increased by adding items to successive trials: Children received one 1-house trial, two 2-house trials, two 3-house trials, and two 4-house trials. Responses were summarized as the number of items answered correctly within each item set.

##### Pick the picture game (working memory)

This is a self-ordered pointing task in which children were presented with a series of 2, 3, 4, and 6 pictures and instructed to continue picking pictures until each picture had “received a turn.” Children are presented with successive pages in which the set of pictures within an item set is re-ordered. The ordering of pictures within each item set is randomly changed (including some trials not changing) so that spatial location is not informative. This task requires working memory because children have to remember which pictures in each item set they have already touched.

##### Silly sounds stroop (inhibitory control)

This task was modeled after the Day–Night Stroop task. Children were asked to make the sound opposite of that associated with pictures of dogs and cats (e.g., meow when shown a picture of a dog).

##### Spatial conflict arrows (inhibitory control)

Children were given two response cards (“buttons”) and were instructed to touch the card consistent with the direction in which an arrow presented on the flipbook page was pointing. Training trials presented compatible images on the same side, and test trials presented arrows contralateral to the correct response (e.g., an arrow pointing right was presented on the left side).

##### Animal go/no-go (inhibitory control)

This is a standard go no-go task in which children were instructed to push a button (which emitted a sound) whenever they saw an animal appear, except when the animal was a pig. The number of go-trials before a no-go trial varied, in a standard order, of 1-go, 3-go, 3-go, 5-go, 1-go, 1-go, and 3-go trials.

##### Something’s the same game (attention shifting)

Children were shown two pictures that were similar on a single criterion (e.g., the same color; the same size), and were then shown a third picture, similar to one of the first two pictures along a second dimension of similarity (e.g., shape). Participants were asked to identify which of the first two pictures was the same as the new picture.

##### Executive function task scoring and composite function

Item response theory (IRT) scoring was used for all tasks in the EF battery. *Z*-scores were calculated to reflect accuracy on each of the six EF assessments. The total score reflected the mean of all completed *z*-scored individual scores. We use a formative composite, as it has been found to more appropriately represent the overarching construct of EF than a latent factor, which is limited to measurement of the shared variance between tasks which are only weakly- to moderately correlated (Willoughby et al., 2016). Prior investigations using the described battery of assessments with the same population have demonstrated acceptable psychometric properties of the resulting EF score (Willoughby et al., 2012). As is typical of EF measures (Willoughby et al., 2014), the reliability coefficient for the composite was relatively low, α = 0.50.

#### Woodcock–Johnson III Tests of Achievement

The Woodcock–Johnson III Tests of Achievement (Woodcock et al., 2001) are a set of co-normed tests that measure general scholastic ability, oral language, and academic achievement and are appropriate for administration for ages 3–92. The reliability and validity of these measures have been well established elsewhere (Woodcock et al., 2001). For all subtests, age-normed standard scores were used.

##### Applied Problems

The Applied Problems (AP) subtest measures early math skills including counting, measurement, and verbal and non-verbal arithmetic and operations.

##### Brief Reading Cluster

The Brief Reading Cluster (BFR) reflects the average of children’s scores on two Woodcock–Johnson subtests: Letter-Word Identification and Passage Comprehension. The Letter-Word Identification (LW) subtest measures basic literacy skills including letter recognition, letter sounds, and reading ability. The Passage Comprehension (PC) subtest also measures basic literacy skills including children’s ability to provide the missing word for a sentence so that it makes sense.

#### Covariates

Individual- and family-level covariates were included in final models of analyses. These covariates included indicator variables for child sex (1 = male; 0 = female), as well as continuous variables for cumulative risk, processing speed, general intelligence, and receptive vocabulary.

##### Cumulative risk

The cumulative risk variable is a mean composite of *z*-scored variables collected from home visits between when participants were 6 and 36 months of age. The variable is made up of items that include family income-to-need ratio (i.e., family income divided by the federal poverty threshold for a family of the relevant size), maternal education, maternal working hour, household density, and a rating of safety of the neighborhood in which the child lives.

##### Processing speed

At the PreK visit, processing speed was measured using two subtests of Wechsler Preschool and Primary Scales of Intelligence (WPPSI; Wechsler, 2002). For assessment of processing speed, the symbol search and coding subscales were used. The symbol search subtest asks participants to scan a group as quickly as possible and indicate whether a target symbol matches any symbols in the group. The coding subscale asks participants to match symbols with geometric shapes, and to reproduce the geometric shapes corresponding to the appropriate symbols.

##### General intelligence

At the age 3 home visit, children completed the block design and receptive vocabulary subtests of the Wechsler Preschool and Primary Scales of Intelligence (WPPSI; Wechsler, 2002). A full-scale IQ score was estimated.

##### Receptive vocabulary

At the PreK visit, receptive vocabulary was measured using the Peabody Picture Vocabulary Test-4th Edition (PPVT; Dunn and Dunn, 2007), a norm-referenced assessment commonly used with children of this age. In this direct assessment, the child is shown four pictures, and the data collector asks the participant to point to one of the four images (e.g., “Can you point to the ball?”). Age-normed standard scores were used in all analyses.

### Analytic Strategy

Our primary research question asks whether EF skills before kindergarten entry uniquely predict academic skills over and above earlier academic skills themselves. Simultaneous models were estimated in a path analysis to regress 5th grade math and reading scores onto EF, PreK math and pre-literacy skills, and other covariates measured prior to kindergarten entry. Next, we sought to investigate whether having high levels of EF prior to kindergarten would buffer against having lower academic skills. Two interaction terms between EF and PreK math and pre-literacy skills were added to the path model. Simple slopes of significant interaction terms were assessed. All models were estimated using Mplus (Muthén and Muthén, 2007) and took the complex sampling design of the Family Life Project into account with sample weights and stratification. In all models, coefficients represent the unique variance attributable to each variable, adjusted for all other variables in the model. Correlations between outcome variables and between predictor variables were estimated in all models.

All analyses are limited to children for whom a direct assessment of EF or academic skills was conducted. Thirteen children were excluded from analyses for having no available direct assessment data, leaving a total of 1,279 participants. For those participants who completed at least one wave of direct assessment, missing data was accounted for using Full Information Maximum Likelihood estimation. Full Information Maximum Likelihood estimation takes into account the covariance matrix for all available data on the independent variables to estimate parameters and standard errors. This approach provides more accurate estimates of regression coefficients than do listwise deletion or mean replacement (Enders, 2001).

## Results

### Descriptive Statistics

Unweighted descriptive statistics and correlations for all variables in the analyses are presented in **Table 1**. *N* = 1026 participants completed EF assessment at age 5; *N* = 877 completed academic ability assessments during their 5th grade year. Standard scores for 5th grade math and reading assessments were near average for the normative sample (Normative Sample: *M* = 100, *SD* = 15; Present Sample: *M* = 98.82, *SD* = 14.94; *M* = 97.86, *SD* = 14.28, respectively). Participants who were not assessed at 5th grade did not differ from those who were assessed at 5th grade on measures of IQ, *t*(1033) = 1.323, *p* = 0.186; speed of processing, *t*(844) = -0.012, *p* = 0.990; receptive vocabulary, *t*(962) = 0.278, *p* = 0.781; EF, *t*(1024) = -0.352, *p* = 0.725; cumulative risk, *t*(1220) = -0.151, *p* = 0.880; or PreK literacy skills, *t*(979) = 1.594, *p* = 0.111. Participants who were assessed at 5th grade scored, on average, 1.5 points higher on the PreK math assessment, *t*(976) = 2.189, *p* = 0.029.

**Table 1:** Descriptive statistics.

	Mean	*SD*	*N*	Range
Age at time of 5th grade testing	11.14	0.40	877	10.25–12.42
Applied Problems score PreK	100.24	12.88	978	29–141
Applied Problems score 5th grade	96.82	14.94	875	1–152
Letter-Word score PreK	98.25	13.29	981	60–156
Brief Reading Cluster score 5th grade	97.87	14.28	876	1–136
PPVT receptive vocab standard score	93.90	15.87	964	43–138
WPPSI speed of processing	96.04	12.61	850	65–133.5
WPPSI IQ	93.57	16.51	1035	45–142
Cumulative risk	0.00	0.69	1222	-2.66–2.19
EF mean score	0.29	0.48	1026	-1.98–1.40

Bivariate correlations for all variables included in the sample are presented in **Table 2**. Academic outcome measures (5th grade math and reading skills) were highly correlated with one another (*r* = 0.701, *p* < 0.001). Additionally, 5th grade scores were highly correlated with pre-kindergarten EF (Math: *r* = 0.506, *p* < 0.001; *r* = 0.435, *p* < 0.001), respectively. Both constructs were also correlated with speed of processing (Math: *r* = 0.398, *p* < 0.001; Reading: *r* = 0.353, *p* < 0.001) and receptive vocabulary (Math: *r* = 0.528, *p* < 0.001; Reading: *r* = 0.513, *p* < 0.001). As such, these direct assessment measures from pre-kindergarten entry were included in all analyses.

**Table 2:** Correlations among variables.

		Correlations								
		1	2	3	4	5	6	7	8	9
(1)	Male	–								
(2)	Age at time of 5th grade testing	0.062	–							
(3)	Applied Problems PreK	-0.061	-0.190***	–						
(4)	Applied Problems 5th grade	0.028	-0.201***	0.630***	–					
(5)	Letter-Word PreK	-0.083**	-0.265***	0.563***	0.432***	–				
(6)	Brief Reading Cluster 5th grade	-0.105**	-0.326***	0.537***	0.701***	0.509***	–			
(7)	Receptive vocab standard score	-0.045	-0.136***	0.664***	0.528***	0.464***	0.513***	–		
(8)	WPPSI speed of processing	-0.185***	-0.223***	0.471***	0.398***	0.423***	0.353***	0.423***	–	
(9)	Cumulative risk	-0.032	0.104**	-0.435***	-0.398***	-0.358***	-0.361***	-0.489***	-0.324***	–
(10)	EF mean score	-0.128***	-0.137***	0.502***	0.506***	0.335***	0.435***	0.533***	0.381***	-0.330^∗∗∗^

*^∗∗^*p* < 0.01, ^∗∗∗^*p* < 0.001*.

### Prekindergarten Predictors of Elementary School Math in 5th Grade

Results of the associations of predictor variables with 5th grade math ability are reported in Model 1 of **Table 3**. Prekindergarten math, and EF were both significantly and uniquely associated with later math ability (β = 0.367, *p* < 0.001; β = 0.209, *p* < 0.001, respectively). Additionally, child sex was associated with late elementary math ability such that, on average, male participants had higher scores than their female peers (β = 0.146, *p* < 0.001). Further, both IQ and processing speed were positively associated with late elementary math ability (β = 0.114, *p* = 0.004; β = 0.081, *p* = 0.028). Receptive vocabulary, cumulative risk, and PreK pre-reading skills were not associated with 5th grade math, net of other variables in the model. As well, 5th grade reading ability remained moderately correlated with math ability (*r* = 0.497, *p* < 0.001). The total model accounted for 53.2% of the total variance in 5th grade math ability scores (*R*^2^ = 0.532).

**Table 3:** Models predicting Applied Problems scores.

	Model 1				Model 2			
	Beta	*SE*	*p*-value	Significance	Beta	*SE*	*p*-value	Significance
AP PreK	0.367	0.041	<0.001	^∗∗∗^	0.439	0.052	<0.001	^∗∗∗^
LW PreK	0.059	0.035	0.097		0.030	0.041	0.471	
Male	0.146	0.027	<0.001	^∗∗∗^	0.143	0.027	<0.001	^∗∗∗^
Receptive vocab	0.045	0.044	0.303		0.038	0.043	0.378	
Processing speed	0.081	0.037	0.028	^∗^	0.077	0.036	0.034	^∗^
IQ	0.116	0.041	0.004	^∗∗^	0.117	0.040	0.004	^∗∗^
Cumulative risk	-0.024	0.032	0.460		-0.033	0.031	0.295	
EF mean score	0.209	0.038	<0.001	^∗∗∗^	0.593	0.246	0.016	^∗^
Age	-0.042	0.026	0.106		-0.043	0.027	0.156	
EF^∗^AP PreK					-0.805	0.284	0.005	^∗∗^
EF^∗^LW PreK					0.390	0.258	0.130	

*^∗^*p* < 0.05, ^∗∗^*p* < 0.01, ^∗∗∗^*p* < 0.001; AP, Applied Problems subtest of the Woodcock–Johnson; LW, Letter-Word Identification subtest of the Woodcock–Johnson*.

### Prekindergarten Predictors of Elementary School Reading in 5th Grade

Results of the simultaneous regression of 5th grade reading on predictor variables are reported in Model 1 of **Table 4**. PreK math, pre-literacy, and EF were significantly and uniquely associated with later reading ability (β = 0.157, *p* = 0.002; β = 0.236, *p* < 0.001; β = 0.149, *p* < 0.001, respectively). Additionally, pre-kindergarten receptive vocabulary was positively associated with later reading (β = 0.128, *p* = 0.004); however, IQ, processing speed, and cumulative risk were not associated with later reading skills. The total model accounted for 44% of the total variance in 5th grade reading scores (*R*^2^ = 0.440).

**Table 4:** Models predicting Brief Reading Cluster scores.

	Model 1				Model 2			
	Beta	*SE*	*p*-value	Significance	Beta	*SE*	*p*-value	Significance
AP PreK	0.157	0.05	0.002	^∗∗^	0.238	0.065	<0.001	^∗∗∗^
LW PreK	0.236	0.043	<0.001	^∗∗∗^	0.219	0.054	<0.001	^∗∗∗^
Male	-0.007	0.029	0.803		-0.011	0.029	0.706	
Receptive vocab	0.128	0.05	0.011	^∗^	0.117	0.049	0.017	^∗^
Processing speed	-0.005	0.036	0.893		-0.007	0.035	0.947	
IQ	0.081	0.047	0.087		0.086	0.047	0.065	
Risk	-0.034	0.041	0.404		-0.045	0.039	0.255	
EF	0.149	0.039	<0.001	^∗∗∗^	0.740	0.277	0.008	^∗∗^
Age	-0.173	0.03	<0.001	^∗∗∗^	-0.169	0.030	<0.001	^∗∗∗^
EF^∗^AP PreK					-0.837	0.353	0.018	^∗^
EF^∗^LW PreK					0.202	0.310	0.255	

*^∗^*p* < 0.05, ^∗∗^*p* < 0.01, ^∗∗∗^*p* < 0.001; AP, Applied Problems subtest of the Woodcock–Johnson; LW, Letter-Word Identification subtest of the Woodcock–Johnson*.

### Does Early EF Buffer against Low Academic Math Skills?

To test whether high levels of early EF would buffer against low levels of early academic skills, we added interaction terms of both EF with PreK math and EF with PreK pre-literacy scores to path model above. Results are reported in Model 2 of **Tables 3**, **4**. The interaction term of EF with PreK math significantly predicted both 5th grade math (β = -0.585, *p* = 0.016) and 5th grade reading skills (β = -0.754, *p* = 0.007), but the interaction term of EF with PreK reading did not relate to either 5th grade outcome.

Inclusion of the interaction terms in the model slightly improved the amount of variance being explained in both 5th grade math (*R*^2^ = 0.544) and reading (*R*^2^ = 0.454). To isolate the moderating role of EF and test whether children who demonstrated different levels of cognitive skills more generally saw differential magnitudes of associations of PreK math and later academic skills, we also tested interactions of PreK math with IQ, processing speed, and receptive vocabulary. None of the resulting interaction effects were significant. For all subsequent analyses, the interaction between EF and PreK reading was removed to better interpret simple slopes.

**FIGURE 1:**
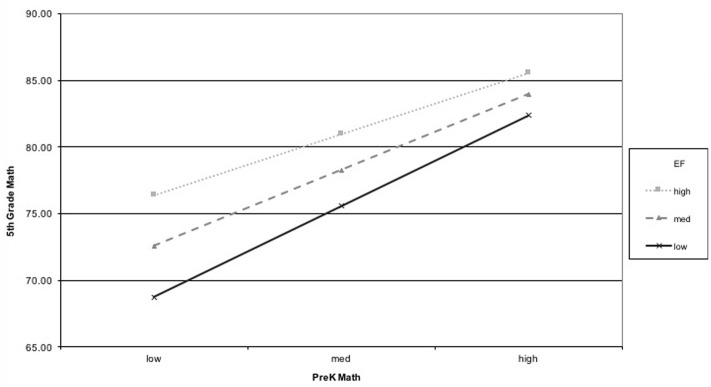
Interaction of executive function (EF) and PreK math predicting 5th grade math.

**FIGURE 2:**
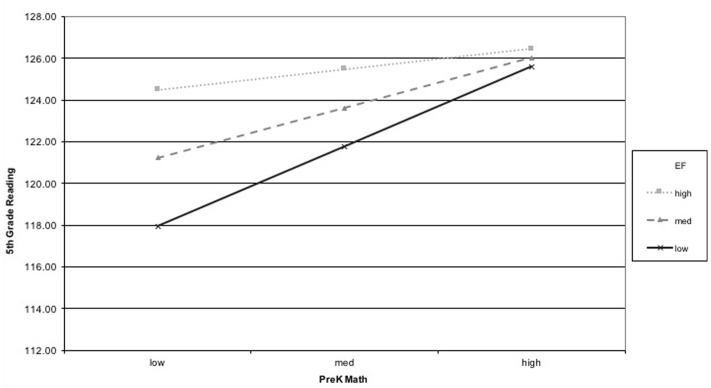
Interaction of EF and PreK math predicting 5th grade reading.

Analysis of simple slopes revealed that for children who at the sample mean for EF, there was a moderate association of PreK math with 5th grade math (β = 0.426, *p* < 0.001) and reading (β = 0.234, *p* < 0.001). For participants who had scores 1SD above the sample mean on EF (e.g., a value of 0.77 on the EF score that reflects the mean of *z*-scores from each individual EF measure, *M*_EF_ = 0.29) the coefficient for PreK math was smaller than that of the sample mean in predicting 5th grade math (β = 0.356, *p* < 0.001) and reading (β = 0.146, *p* = 0.001). Similarly, the simple slopes for values 1SD below the sample mean for EF (e.g., a value of -0.19) were also significant, such that children who had low levels of EF in PreK had a higher magnitude of the coefficient on pre-kindergarten Applied Problems scores for 5th grade math (β = 0.509, *p* < 0.001) and reading (β = 0.330, *p* < 0.001). In other words, the variance of 5th grade math and reading ability associated with earlier math changed as a function of children’s EF, such that children with a higher level of EF ability at PreK were better able to “catch up” with their peers who were better at math in PreK. This is shown graphically in **Figures 1**, **2**.

## Discussion

The goal of this study was to investigate the role of EF in predicting academic achievement in late elementary school in a diverse sample of children from low-income families. In particular, we were interested in whether there was an association between EF and 5th grade math and reading achievement over and above the predictive value of earlier math and reading scores and other cognitive abilities. We also sought to investigate whether the predictive value of PreK math and reading abilities varied as a function of child EF.

In our analysis of main effects only, we found that, while early math and reading were both important predictors of later math and reading, PreK EF was associated with more than 1/5th of a standard deviation in math (three points on the standardized measure of 5th grade math used in the present sample), and nearly 1/7th of a standard deviation in reading (over two points on the reading measure). This association was net of other cognitive covariates, including IQ (1/10th of a standard deviation in math), processing speed (1/12th of a standard deviation in math), and receptive vocabulary (1/8th of a standard deviation in reading), and the predictive value of EF was greater than that of other cognitive covariates.

In testing the interaction between EF and early academic ability, we found a significant interaction between EF and early math (but not EF and pre-reading skills) indicating that higher EF ability can compensate to some extent for limited academic knowledge prior to school entry. Children with initially low math ability but with higher EF may still reach the levels of achievement in math and reading typically associated with more proficient domain-specific prerequisite skills. This suggests EF may serve as an important skill set that helps students “catch up” with their higher-achieving peers in academic settings, even if they start out behind.

Notably, prior investigations (e.g., Duncan et al., 2007; Pagani et al., 2010) have suggested early math is a stronger predictor of later reading than early pre-reading or other skills. In the current investigation, we find there is a domain-specific relation between early and later skills: pre-reading is the strongest predictor of later reading skills, as early math is the strongest predictor of later math skills. In fact, in the analyses predicting 5th grade reading scores, both EF and receptive vocabulary predict between 13 and 15% of a standard deviation in later reading scores, which is comparable to the 16% of a standard deviation predicted by early math ability.

Altogether, our results suggest one major theme: early EF is important in the development of later academic skills. Not only is EF a unique predictor of 5th grade math and reading ability, but our analysis suggests that high levels of early EF can help to compensate for low levels of academic ability in PreK. This interaction between math and EF in PreK is of particular interest and merits additional investigation. This finding in the present study extends prior analyses from this dataset demonstrating that EF moderates the magnitude of the association between PreK and kindergarten math (Blair et al., 2016). If indeed there is a group of children who had high EF who performed on par with their peers who were more proficient in math in PreK in 5th grade skills, this may support other empirical evidence that suggests intervention on early EF is important for success in school. A person-centered analysis may shed light on this.

The relation between PreK math and 5th grade reading, as well as the relation between the interaction of PreK math and EF and 5th grade reading merits additional discussion. As was suggested by Duncan et al. (2007), the association between early math and later reading may be spurious, despite extensive and robust controls for home and individual cognitive characteristics; however, as this finding has now been replicated in multiple large, prospective datasets (e.g., Duncan et al., 2007; Pagani et al., 2010), it seems likely there is some signal through the noise. Importantly, in the present investigation and others that have found the association between early math and later reading, the assessment of early mathematical skills privileges word problems, which are read to the participant by a trained assessor. In order to correctly solve each problem, participants must understand the demands of the task, decode what the problem is asking them to do, compute and discover the response, and respond in an appropriate way. These steps require engagement of EF and are, in many ways, also central to reading comprehension. In contrast, the assessment of early reading skills in the present investigation requires knowledge of letter words and sounds, which is an important facet of learning to read, but is less relevant for children once they make the transition to reading to learn.

Ultimately, the present investigation contributes to the growing literature about the role of EF in education. Other studies have found EF is a strong and stable predictor of later academic skills (Best et al., 2011). This is an important and provocative finding; however, additional research is needed to better understand the mechanisms by which EF contributes to the development of academic skills. Various hypotheses have been tested and have suggested there may be a role of EF in fostering positive relationships with teachers (Blair et al., 2016) and in promoting self-regulatory behaviors in the service of learning from instruction in the classroom (Brock et al., 2009), or completing homework outside the classroom (Langberg et al., 2013). It is likely a combination of these and other skills that serve as mediating mechanisms by which EF affects academic learning. These may also account for the finding here that high levels of EF serve as a way for children to “catch up” even though they have low levels of math ability in PreK: Children who are able to leverage their high levels of EF to be more engaged, attentive, and productive inside and outside the classroom may ultimately learn more material. Separately, it may be that higher levels of academic skills promote the development of EF, as has been found previously (Daneri and Blair, 2017). Further research is needed to better understand both the uni-and bi-directional relations between EF so as to better intervene upon on and foster the development of EF in young children.

The role of EF in the development of math skills is well established. This study is consistent with the findings of a number of prior analyses, which suggest early EF is related to math ability throughout the academic lifespan (Mazzocco and Kover, 2007; Bull and Lee, 2014), and that having high levels of at least some aspects of EF (e.g., working memory) may be associated with faster growth in math ability (Lee and Bull, 2016). This study is somewhat unique, however, in controlling for a host of covariates, including academic knowledge and ability measured prior to school entry as well as multiple highly robust correlates of both EF and academic achievement, namely processing speed, receptive vocabulary, and general intelligence. Notably, in this analysis, the magnitude of the association on EF was greater in predicting math than reading skills in the 5th grade. Several prior studies have also found this to be the case (Best et al., 2011). The role of EF, particularly working memory, in reading and vocabulary, however, is well established (e.g., Daneman and Carpenter, 1980; Alloway, 2010; Loosli et al., 2012; Karbach and Verhaeghen, 2014). In part, associations between EF and reading are attributable to a close association between EF and language development (Gathercole and Baddeley, 1989; Daneman and Merikle, 1996). Specific effects of EF on reading are seen most consistently for reading comprehension and fluency, however, rather than for more basic, knowledge-based aspects of reading, such as knowledge of letters and words (Cutting et al., 2009; Sesma et al., 2009; Kieffer et al., 2013; Fuchs et al., 2015). The Woodcock–Johnson Brief Reading Cluster analyzed here combines the letter-word subtest with the passage comprehension subtest and it may be that this more knowledge-based aspect of the assessment led to a reduced association between EF and reading. This may also have important implications as to why we do not find a significant interaction of PreK Letter-Word scores and EF: it may be that the variance explained in 5th grade Brief Reading Cluster scores by letter-word scores is unrelated to EF, and that variance attributable to EF is limited to the passage comprehension. It may also be that the relation of EF to mathematics is in fact stronger in the elementary grades.

Of additional interest, our results reveal an association of child gender with scores on math, but not reading. Extensive research has suggested a correlation between cultural beliefs of gender stereotypes in academic performance and the realized gender-based gap in performance on math and science on an international level (Nosek et al., 2009). Indeed, within the United States, there has long been documented strong cultural stereotype that math is a male domain (Hyde et al., 1990). These beliefs are embedded in our daily lives and can be seen both implicitly and explicitly in children as early as elementary school (Lummis and Stevenson, 1990; Cvencek et al., 2011). Active efforts are being made to better understand how gender stereotypes about academic achievement are communicated to young children (Gunderson et al., 2012), and to intervene on and mitigate the effects of the cultural embeddedness of gender stereotypes in math and science fields (Beede et al., 2011).

There are several limitations that must be addressed in the context of this investigation. First, it is important to note that while this study is longitudinal in nature, causality cannot be inferred. Second, there is a large literature that has described the importance of teacher, school, and classroom characteristics in the development of early academic skills (including math, reading, and EF) and growth in those academic skills throughout schooling. In the present study, we lack measurement of instructional quality and school and classroom context. These are important omitted variables that may account for additional variance in outcome measures. Additionally, the present sample is limited to only two regions of the United States, and results may not generalize to others or to regions outside the United States. The current findings may only apply to children from rural areas of the United States, or to children born to low-income families. Finally, it is important to note that assessment of academic skills was limited to research assistant administered standardized assessments. While performance on these assessments is generally correlated with performance on formative and summative assessments in school contexts, it is likely these assessments capture only some aspects of math and reading achievement. Finally, it is important to note that the measurement of both EF and math and reading is complex, and though we use well-established and comprehensive measures, there remains aspects of those constructs that go unmeasured. For example, one of our assessments of EF assesses aspects of short term memory in addition to working memory, and working memory cannot be isolated. Similarly, the assessment of math ability privileges certain aspects of mathematics knowledge (e.g., counting, cardinality, and operations) over others (e.g., geometry).

Despite these limitations, results from the present investigation make a strong case for the importance of early skills. Beyond math and reading, there should be a focus in early childhood education on the development of EF, as EF fosters the development of high level math and reading in late elementary school, and may even serve as a mechanism by which children can catch up to their high achieving peers.

## Ethics Statement

This study was carried out in accordance with the recommendations of the Institutional Review Board at Pennsylvania State University and the Office of Human Research Ethics at the University of North Carolina with written informed consent from all subjects. All subjects gave written informed consent in accordance with the Declaration of Helsinki. The protocol was approved by the Institutional Review Board at Pennsylvania State University and the Office of Human Research Ethics at the University of North Carolina.

## Author Contributions

AR conceptualized the study, carried out the initial analyses, drafted the initial manuscript, and approved the final manuscript as submitted. CB and MW reviewed and revised the manuscript, and approved the final manuscript as submitted.

## Conflict of Interest Statement

The authors declare that the research was conducted in the absence of any commercial or financial relationships that could be construed as a potential conflict of interest.
